# Postural Changes in Blood Pressure Associated with Interactions between Candidate Genes for Chronic Respiratory Diseases and Exposure to Particulate Matter

**DOI:** 10.1289/ehp.0800279

**Published:** 2009-02-03

**Authors:** Elissa Wilker, Murray A. Mittleman, Augusto A. Litonjua, Audrey Poon, Andrea Baccarelli, Helen Suh, Robert O. Wright, David Sparrow, Pantel Vokonas, Joel Schwartz

**Affiliations:** 1 Department of Environmental Health and; 2 Department of Epidemiology, Harvard School of Public Health, Boston, Massachusetts, USA; 3 Channing Laboratory, Brigham and Women’s Hospital, Harvard Medical School, Boston, Massachusetts, USA; 4 Center for Molecular and Genetic Epidemiology, Department of Environmental and Occuptional Health, University of Milan, Milan, Italy; 5 IRCCS Maggiore Hospital, Mangiangalli and Regina Elena Foundation, Milan, Italy; 6 Veterans Administration Normative Aging Study, Veterans Administration Boston, Department of Medicine, Boston University School of Medicine, Boston, Massachusetts, USA

**Keywords:** aging and susceptible populations, blood pressure, environmental epidemiology, gene-environment interaction, particulate matter

## Abstract

**Background:**

Fine particulate matter [aerodynamic diameter ≤ 2.5 μm (PM_2.5_)] has been associated with autonomic dysregulation.

**Objective:**

We hypothesized that PM_2.5_ influences postural changes in systolic blood pressure (ΔSBP) and in diastolic blood pressure (ΔDBP) and that this effect is modified by genes thought to be related to chronic lung disease.

**Methods:**

We measured blood pressure in participants every 3–5 years. ΔSBP and ΔDBP were calculated as sitting minus standing SBP and DBP. We averaged PM_2.5_ over 48 hr before study visits and analyzed 202 single nucleotide polymorphisms (SNPs) in 25 genes. To address multiple comparisons, data were stratified into a split sample. In the discovery cohort, the effects of SNP × PM_2.5_ interactions on ΔSBP and ΔDBP were analyzed using mixed models with subject-specific random intercepts. We defined positive outcomes as *p* < 0.1 for the interaction; we analyzed only these SNPs in the replicate cohort and confirmed them if *p* < 0.025 with the same sign. Confirmed associations were analyzed within the full cohort in models adjusted for anthropometric and lifestyle factors.

**Results:**

Nine hundred forty-five participants were included in our analysis. One interaction with rs9568232 in PHD finger protein 11 (*PHF11*) was associated with greater ΔDBP. Interactions with rs1144393 in matrix metalloprotease 1 (*MMP1*) and rs16930692, rs7955200, and rs10771283 in inositol 1,4,5-triphosphate receptor, type 2 (*ITPR2*) were associated with significantly greater ΔSBP. Because SNPs associated with ΔSBP in our analysis are in genes along the renin–angiotensin pathway, we then examined medications affecting that pathway and observed significant interactions for angiotensin receptor blockers but not angiotensin-converting enzyme inhibitors with PM_2.5_.

**Conclusions:**

PM_2.5_ influences blood pressure and autonomic function. This effect is modified by genes and drugs that also act along this pathway.

Exposure to fine particulate matter [aerodynamic diameter ≤ 2.5 μm (PM_2.5_)] has been associated with cardiovascular outcomes, including myocardial infarctions (Peters et al. 2001; Tonne et al. 2007; Zanobetti and Schwartz 2005), endothelial dysfunction (O’Neill et al. 2007), and electrocardiographic ST segment depression (Gold et al. 2000), but the mechanisms responsible for these associations have not been not fully elucidated. Several epidemiologic studies have reported associations between PM air pollution and autonomic changes measured by heart rate variability (Chuang et al. 2007; Luttmann-Gibson et al. 2006; Schwartz et al. 2005) and have shown that this association is modified by genetic polymorphisms (Chahine et al. 2007). Air pollution has also been associated with vasoconstriction (Brook et al. 2002) and increases in blood pressure (Ibald-Mulli et al. 2001), suggesting effects on blood pressure control.

Postural change in blood pressure is a measure of cardiovascular reactivity reflecting autonomic function indicated by baroreflex-mediated feedback. Movement from a supine or sitting position to standing causes a rapid loss of blood from the thoracic and abdominal cavities and pooling in the extremities, reducing venous return and cardiac stroke volume. Under normal conditions, this stimulates baroreceptors to activate the sympathetic nervous system, leading to vasoconstriction and increased heart rate to maintain a stable blood pressure as parasympathetic nerve signals to the heart are withdrawn, thus causing short-term blood pressure changes. Although up-regulation of sympathetic activity is necessary for regulation of blood pressure, hyperreactivity is associated with harmful effects, including the development of hypertension.

Because chronic lung disease is associated with many processes also involved with cardiovascular disease, we hypothesized that genes originally selected for study of chronic obstructive pulmonary disease (COPD) and asthma would influence postural change in blood pressure and interact with effects of PM on this measure. Asthma and COPD are well-established diseases of immune response, and evidence has linked these conditions with the autonomic and immune systems (Mignini et al. 2003) in addition to demonstrating that the renin–angiotensin system is activated in severe asthma (Ramsay et al. 1997). Polymorphic variation may make some individuals more vulnerable to the effects of PM exposure, and PM has been associated with increases in systolic blood pressure (SBP) (Ibald-Mulli et al. 2001). Therefore, we predicted that these interactions may play a role in postural blood pressure changes as well.

## Materials and Methods

### Study population

This study uses data from the Normative Aging Study (NAS), details of which have been published previously (Bell et al. 1970). Briefly, the NAS is an ongoing longitudinal cohort study of aging established by the Veterans Administration in 1963. A total of 2,280 men from the Greater Boston area (21–80 years of age) confirmed to be free of known chronic medical conditions were enrolled, and participants were asked to return for examinations every 3–5 years. Study center visits took place in the morning, after overnight fast and abstention from smoking. Physical examinations included measurement of height and weight, and body mass index (BMI) was calculated as weight (in kilograms) divided by height (in square meters). Questionnaires evaluated smoking habits and medication use, with responses confirmed by an on-site physician. Subjects indicated pulmonary disorders by a questionnaire based on the American Thoracic Society Division of Lung Diseases 1978 questionnaire (Ferris 1978). Participants provided written informed consent, and the study protocol was approved by the institutional review boards of all participating institutions. Subjects visited the study center during the study period from 1995–2006 one to four times: 945 subjects (45%) had one visit, 692 subjects (33%) had two visits, 425 subjects (20%) had three visits, and 36 subjects (2%) had four visits.

### Blood pressure measurements

At each clinical visit, a physician measured blood pressure using a standard mercury sphygmomanometer. SBP and fifth-phase diastolic blood pressure (DBP) were measured in each arm to the nearest 2 mmHg while the participant was seated. The means of the right and left arm measurements were used as each participant’s SBP and DBP. A measure of standing blood pressure was recorded in the right arm 30 sec after standing. Previous studies have noted that most hemodynamic changes related to the assumption of standing posture occur within the first 30 sec (Akselrod et al. 1997; Smith et al. 1994), and the NAS protocol was designed to address these initial orthostatic changes. Postural changes in SBP and DBP were calculated as mean sitting minus standing for SBP (ΔSBP) and DBP (ΔDBP).

### Air pollution measurement

Ambient PM_2.5_ were measured beginning in 1995 on the roof of Countway Library at Harvard Medical School. PM_2.5_ concentrations were measured using Tapered Element Oscillating Microbalance (model 1400A; Rupprecht & Pasternack, Albany, NY) operated at 50°C with a PM_2.5_ impactor (Air Diagnostics, Harrison, ME) with two plates. Air was drawn through the impactors at 4 LPM. Concentrations (microgram per cubic meter) were averaged into 30-min periods. For this study, we estimated exposures as the 48-hr average concentration before study visit and blood draw. This time period has shown to have strongest associations with heart rate variability, another measure of autonomic function, within this cohort (Park et al. 2005).

### Single nucleotide polymorphism selection and genotyping

We chose candidate genes based on their known or suspected roles in the etiology of COPD and asthma from previous positional cloning and candidate gene studies (Poon et al. 2007a, 2007b). A total of 202 single nucleotide polymorphisms (SNPs) in 25 genes were available for this analysis, which took advantage of another project that had extensively genotyped genes suspected of being related to chronic lung disease. SNPs were located in genes associated with lung inflammation, protease/antiprotease imbalance, oxidative stress, and extracellular matrix synthesis or destruction. We also selected tagging SNPs with *r*^2^ > 0.80 and minor allele frequency (MAF) > 5%, covering 5 kb upstream and downstream of the first and last exons of each gene, nonsynonymous amino acid change with MAF > 1%, and known variants associated with asthma and related phenotypes. Table 1 lists the 25 genes associated with these SNPs.

For genotyping we used BeadStation 500G (Illumina Inc., San Diego, CA) in the first stage (discovery cohort) and replicated results either with Sequenom MassArray matrix-assisted laser desorption/ionization time-of-flight mass spectrometer (Sequenom, San Francisco, CA) with semi automated primer design (SpectroDESIGNER, Sequenom) and implementation of the very short extension method (Sun et al. 2000), or with TaqMan 5′ exonuclease with primers from Applied Biosystems Inc. (Foster City, CA) using radioactively labeled probes detected using ABI PRISM 7900 Sequence Detector System (Applied Biosystems Inc.).

Population stratification was assessed using 101 unlinked SNPs chosen from the Celera data set on the SNP Consortium Web site (http://snp.cshl.org/). We tested identity-by-descent clustering using the PLINK tool set version 2 (http://pngu.mgh.harvard.edu/purcell/plink/; Purcell et al. 2007). SNPs included in the stratification panel had Illumina scores ≥ 0.65, indicating a high likelihood of successful genotyping, and a MAF ≥ 0.25 in Caucasians, and did not map to a gene in SNPper (http:/snpper.chip.org/). SNPs fewer than 100 kb apart, or those located on X-chromosome or on chromosomes 2q, 8p, 12p, 19q were not included in the population stratification analysis because these regions are thought to be in linkage for chronic lung disease and, by definition, would be expected to differ within the population based on disease conditions.

### Statistical methods

Multiple comparisons is a major issue in genetic association studies. To address this issue, we used a two-stage strategy, dividing the study population into a discovery cohort and a replication cohort. The rationale for this approach is that random chance can create false associations in either cohort, but because it is random there is no reason to expect the same SNPs to have the same false associations in both cohorts. We defined change in blood pressure as standing minus sitting blood pressure, so that a negative number indicated a drop in blood pressure. Models of ΔSBP and ΔDBP were analyzed using linear mixed effect models with random intercepts for individual subjects; in each model, we included terms for an individual SNP, PM_2.5_, and SNP × PM_2.5_ interaction adjusted for age and BMI. Genotypes (wild-type and heterozygous and homozygous variants) were coded using categorical variables (levels 0, 1, 2) for testing under an additive model, and as binary variables (0, 1) for testing under dominant and recessive models. The entire SNP set was analyzed in the discovery cohort and SNP × PM_2.5_ interactions associated with postural change in blood pressure at the *p* < 0.1 level were selected for testing in the replication cohort. We analyzed only these associations in the replication cohort and confirmed associations that were replicated in the same direction as in the discovery cohort (positive or negative in both data sets) with a *p* < 0.025 in the replication cohort. Confirmed associations were then examined in models adjusted for age, BMI, smoking status (never, current, former), blood urea nitrogen, individual class of hypertension medication use [beta blockers, angiotensin receptor blockers (ARBs), alpha blockers, angiotensin-converting enzyme (ACE) inhibitors, and calcium channel blockers], and diabetes diagnosis in the full cohort data.

The splitting of the sample and use of confirmation in the replication cohort provide protection against false positives. Because of the correlations in the data among SBP and DBP, repeated measures, and SNPs, the exact false discovery rate under this procedure cannot be computed theoretically. Instead, we examined false-positive predictive rates in a simulation study that preserved the correlation structure among SNPs and outcomes present in the data. The genotype data for each subject was randomly permuted, and then merged back into the repeated measures of phenotype. We created 10 new simulation data sets in which any associations observed in each individual simulation were random. We then identified all SNPs within each of the 10 discovery simulations that met our criteria of a *p* < 0.1 for the SNP × PM_2.5_ interaction and analyzed them in the simulated replication data set. We defined the probability of observing each of the associations we saw in the true data set as the probability of observing a greater absolute value *t*-statistic in the replication arm of the simulation study.

## Results

A total of 945 individuals with a total of 2,098 study visits provided complete covariate and genotype data and are included in this analysis. A total of 480 subjects were randomly assigned to the discovery data set and 465 to the replicate data set. Table 2 presents the distribution of their demographics and anthropometrics. Age (mean ± SD) of study participants was 71.0 ± 7.3 years in the discovery cohort and 71.6 ± 7.1 years in the replication cohort, and most participants were former smokers. The distributions of a history of diabetes and use of medications for hypertension were similar in both cohorts.

We first examined the main effects of PM_2.5_ in mixed models adjusted for all covariates. The effect of PM_2.5_ in the model for ΔDBP was associated with a −0.018 mmHg change [95% confidence interval (CI), −0.45 to 0.41]. The main effect of PM_2.5_ in the model for ΔSBP was 0.69 mmHg (95% CI, −0.15 to 1.53). Both of these results are non-significant, although the ΔSBP change was more suggestive of an association.

All of the SNPs that met our criteria for an association in analyses were in Hardy**–**Weinberg equilibrium. Table 3 presents the distribution and Hardy**–**Weinberg equilibrium chi-square test results and *p*-values for SNPs that met the criteria in our models. The rs9568232 SNP in PHD finger protein 11 (*PHF11*) met our criteria for an SNP × PM_2.5_ interaction predictive of ΔDBP. Criteria for SNP × PM_2.5_ interactions predictive of ΔSBP were met by rs114493 in matrix-mettalo-protease 1 (*MMP1*) as well as rs16930692, rs7955200, and rs10771283 in the inositol 1,4,5-triphosphate receptor, type 2 (*ITPR2*).

We examined SNP × PM_2.5_ interactions under additive, dominant, and recessive models of inheritance, and in our ΔDBP models 32 associations in 21 SNPs met criteria for ΔDBP in the discovery data set and were replicated. Of these associations, one SNP in the *PHF11* gene met criteria for a significant SNP × PM_2.5_ interaction. Under a dominant model, the C/T *PHF11* rs9568232 SNP × PM_2.5_ interaction was associated with 2.29 mmHg higher DBP upon standing (95% CI, 0.72–3.86). Neither the main effect of PM_2.5_ (−0.3 mmHg; 95% CI, −0.79 to 0.19) nor rs9568232 (0.37 mmHg; 95% CI, −0.49 to 1.23) was significant in the model.

In our SBP models, 46 associations in 34 SNPs met criteria at the 0.1 level for ΔSBP in the discovery data set. Of these associations, four SNP × PM_2.5_ interactions met criteria for significant changes in ΔSBP (Table 4). Under a recessive model of inheritance, the SNP × PM_2.5_ interaction for the rs1144393 C/T SNP in *MMP1* was associated with a 3.40 mmHg higher ΔSBP (95% CI, 0.79–6.01). Both main effects for the SNP and PM_2.5_ were not significant at the mean level of PM_2.5_ exposure.

Three SNPs in *ITPR2* met criteria for an association with ΔSBP modified by the effect of PM_2.5_. Under a dominant model, the SNP × PM_2.5_ interaction for the minor C allele of A/C rs16930692 was associated with 3.48 mmHg higher SBP upon standing (95% CI, 0.84–6.12). Similarly, under a recessive model, the SNP × PM_2.5_ interaction for the T variant of the C/T rs7955200 SNP in *ITPR2* was associated with a 4.84 mmHg higher SBP upon standing (95% CI, 1.47–8.20). Finally, for the G minor allele of the rs10771283 A/G SNP, we observed 5.7 mmHg higher ΔSBP (95% CI, 1.71–9.69). For all three *ITPR2* SNPs, main effects of the SNPs and PM_2.5_ were not significant.

**Figure 1:**
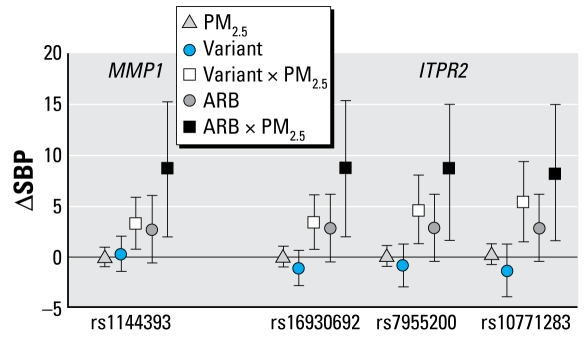
Effect of PM_2.5_, SNPs, Angiotensin receptor blocker (ARB), SNP × PM_2.5_ interactions, and ARB × PM_2.5_ interactions in models predicting ΔSBP. We adjusted all models for age, BMI, smoking status, ACE inhibitors, ARBs, diabetes, alpha blockers, beta blockers, and calcium channel blockers.

Because these ΔSBP associations for SNP × PM_2.5_ interactions were observed in genes related to the renin–angiotensin system, we hypothesized that blood pressure medications such as ARBs and ACE inhibitors might also be modified by the effects of PM_2.5_. We then examined associations for medication × PM_2.5_ interactions in the full cohort by adding interaction terms for medication × PM_2.5_ separately to the models that met our criteria for SNP × PM_2.5_ interactions (Table 4). Significant ARB × PM_2.5_ interactions (*p* < 0.05) were observed for all of the SNPs that met criteria for ΔSBP SNP × PM_2.5_ interactions, which were previously confirmed through our two-stage analysis utilizing the split-sample approach. In these models, the coefficients for the ARB × PM_2.5_ interactions were associated with significantly higher ΔSBP, whereby the magnitude of increase for an increase in 10 μg/m^3^ above the mean level of PM_2.5_ exposure was associated with 8–9 mmHg higher SBP. Similar results were observed for ARB × PM_2.5_ interactions in the absence of any SNP, where the interaction was associated with a 7.16 mmHg higher SBP (95% CI, 1.09–13.22). Figure 1 displays the relationship between SNP × PM_2.5_ interactions and ARB × PM_2.5_ interactions for ΔSBP. ACE inhibitors and their interactions with PM_2.5_ did not significantly alter ΔSBP.

Using our simulations, we found that the adjusted *p*-value for the SNP × PM_2.5_ association observed for the rs9568232 SNP in *PHF11* was 0.01. The rs1144393 *MMP1* SNP × PM_2.5_ interaction associated with ΔSBP had an adjusted *p*-value of 0.02. The adjusted *p*-values for both the rs16930692 and rs10771283 *ITPR2* SNP × PM_2.5_ interactions were 0.012, and for rs7955200 the adjusted *p*-value for the SNP × PM_2.5_ interaction was 0.004.

## Discussion

In this study of the effects of SNP × PM_2.5_ interactions predicting postural changes in blood pressure, we observed several significant associations suggesting autonomic dysregulation and sensitization related to genes acting along the renin–angiotensin pathway in the presence of PM_2.5_, in contrast to the typical physiologic responses, in which we would expect to observe very small perturbations in blood pressure, and even negative associations with initial orthostatic change. Interactions in this analysis were consistently associated with increases in blood pressure from sitting to standing. In models of ΔDBP we observed one SNP × PM_2.5_ interaction that was a significant predictor, whereas in ΔSBP models, a single SNP in *MMP1* and three SNPs in *ITPR2* met our criteria. *ITPR2* and *MMP1* are activated downstream from angiotensin II and may be involved in altering responsiveness to renin–angiotensin and subsequent vasoconstriction with chronic exposure. Our analysis including PM_2.5_ interactions with antihypertension medication use among study participants showed similar effects where both SNP × PM_2.5_ interactions and ARB × PM_2.5_ interactions were associated with greater increase in blood pressure from sitting to standing position.

Although angiotensin II sensitization is necessary for regulation of blood pressure, hyper-reactivity is associated with harmful effects, including the development of hypertension. Recent studies suggest that PM_2.5_ introduces cardiovascular effects by blunting response to endothelial dependent agonists as well as increasing response to vasoconstrictors in animals (Sun et al. 2005). In humans, evidence has shown that PM alters vascular diameter and tone and is associated with narrowing of the brachial artery (Brook et al. 2002) in controlled laboratory settings. Although epidemiologic studies have observed conflicting results in response to the effects of PM_2.5_ on blood pressure, two recent reports have observed small but significant increases in blood pressure associated with exposure (Auchincloss et al. 2008; Zanobetti et al. 2004). Our results suggest that looking at main effects of genes alone may not be sufficient and also may help to explain why associations between PM_2.5_ and various cardiovascular outcomes have been inconsistent.

We observed a number of associations for SNP × PM_2.5_ interactions as predictors of ΔSBP, but only one SNP met criteria in our models for an association with ΔDBP. *PHF11* is located on chromosome 13 and is most commonly cited as an asthma candidate gene. A chromosomal region of 13q4 within which *PHF11* is located has been previously associated with a linkage peak thought to be related to a site controlling both SBP postural change and BMI, a well-established predictor of blood pressure (Feitosa et al. 2002; North et al. 2004). One recent study has shown that asthmatics were 43% more likely to have heart disease and 36% more likely to have high blood pressure compared with nonasthmatics (Dogra et al. 2007), and adult-onset asthma has been demonstrated to be a significant predictor of atherosclerosis (Onufrak et al. 2007). The *PHF11* gene has been associated with IgE regulation, and different splice variants of the gene are differentially expressed in B- and T-lymphocytes (Zhang et al. 2003).

Examination of the SNPs associated with ΔSBP in our models showed that both genes in which these SNPs are located also act along the renin–angiotensin pathway. The rs1144393 SNP in *MMP1* SNP for which we observed an increase in ΔSBP via SNP × PM_2.5_ effects is located in the promoter region of the gene, primarily responsible for expression of the protein. MMPs are involved in extracellular matrix remodeling, which is closely related to mechanisms of atherosclerosis and plaque disruption. Diesel particles are well-established inducers of oxidative stress and have been shown to induce *MMP1* (Amara et al. 2007), providing further support for a functional relationship between the effects of *MMP1* and cardiovascular outcomes. Also, angiotensin II has also been shown to induce expression of *MMP1* in human vascular muscle cells (Kim et al. 2005).

*ITPR2*, for which three SNPs were significant, is expressed widely in myocytes, and expression of these receptors is altered in heart failure, which suggests that the signaling mechanism associated with *ITPR2* is responsible for critical cardiac functionality for contractility through the regulation of Ca^2+^ channels that are important in the maintenance of vascular tone (Kockskamper et al. 2008). Also, angiotensin II is known to affect baroreceptors by releasing intercellular calcium through interaction with inositol triphosphate (Paton et al. 2001) and binds to cell surface receptors, which in turn directly activate *ITPR2* to release intracellular calcium from the endoplasmic reticulum, triggering a further cascade resulting in vasoconstriction. The SNPs in which we observed an association are all located in intronic regions; although the functionality of these variants is not well understood, they may play a role in stability of the protein product, or they may be correlated with SNPs in other regions with significant effects.

Although we noted effect modification between ARBs and PM_2.5_ for both *ITPR2* and *MMP1* SNPs, we observed no relationship for the interaction with ACE inhibitors; this may be due to the site of action of ARBs being more immediately proximal to the triggering of expression of these genes. Although the two classes of drugs ultimately produce similar effects in reducing blood pressure, they work by different mechanisms along the renin–angiotensin pathway. Sun et al. (2008) recently noted that treatment of rats with angiotensin II in conjunction with exposure to air pollution increased aortic vasoconstriction, which is consistent with our observations. Interestingly, Li et al. (1998) found that losartan, an ARB, inhibits the vasoconstriction effect of urban PM on human pulmonary artery endothelial cells.

Although this study has produced a number of novel findings, we recognize that it also has some limitations. This study was not conducted using genomewide association techniques but rather was performed on a subset of genes. We took advantage of another project that had extensively genotyped genes thought to be related to chronic lung disease, based on our belief that heart disease and chronic lung disease share many pathways. This limited our ability to study all the genes we might have been interested in if we had been able to examine genes related to blood pressure and baroreceptor response. However, we believe that the genes chosen for asthma and COPD are plausible candidates for these measures of autonomic function. We also considered the possibility that we observed our significant interactions because of associations between the sitting SBP, DBP, and mean arterial pressure effects alone, but analysis confirmed no significant association for any of the SNPs that met our criteria. These SNPs also were not associated with antihypertension medication use.

Although the ambient PM_2.5_ concentrations used in this analysis were obtained from a single monitoring site, because of the spatially homogeneous nature of PM_2.5_ and previous evidence from a recent study demonstrating high longitudinal correlation between area concentrations and personal exposures (Sarnat et al. 2005), we believe that the single-site PM_2.5_ measurements should serve as a reasonable surrogate. In addition, misclassification of the air pollution monitoring data would be expected to be nondifferential and thus attenuate the true PM_2.5_ effect. Measurement error in our blood pressure monitoring data would likely have a similar nondifferential effect, thereby decreasing the likelihood of statistical significance. As a result, we could have missed some true associations between SNPs and biomarkers because our statistical approach is more sensitive to false positives than to false negatives.

We have used a simple approach to measuring autonomic function, and we have adopted stringent criteria for replication and calculated adjusted *p*-values using a permutation approach, allowing us to preserve the correlation structure within the data. We adopted this strategy rather than the classic Bonferroni correction method because the latter is not appropriate for testing associations within complex correlation structures and is designed to overlook true associations at the expense of identifying spurious ones. Other procedures such as the widely used Benjamini and Hochberg (1995) false discovery rate use a modified Bonferroni approach to be less conservative, but even these tests generally assume independence or only weak correlation. In this analysis, there is correlation between the postural change in blood pressure measurements and the repeated measures of the blood pressure measurements within each subject. Additionally, the density of SNPs selected within genes for this analysis suggests high probability of genetic linkage, and it is possible that some of the associations we observed may be markers of other functional regions. Correlation also exists because we have conducted tests under additive, dominant, and recessive models of inheritance. The use of a simulation approach is a simple yet comprehensive way to address multiple testing.

## Conclusions

In this study of the effects of PM_2.5_ interactions with asthma and COPD candidate genes and hypertension medication use on ΔSBP and ΔDBP, we observed significant effect modification by PM for several genes and for ARB use. Because we have examined these associations under a situation in which we are using outcomes measured on a continuous scale and with repeated measures, we have increased power to detect novel associations that might otherwise go unobserved. Response to air pollution and its effect on autonomic function may be mediated by the SNP variants we have identified and can be further investigated in case–control and cohort studies. Also, blunting of the effects of ARBs but not ACE inhibitors in response to air pollution merits increased attention in clinical settings.

## Figures and Tables

**Table 1:** Candidate genes included in our analysis.

Gene	Name	Chromosome position
*ITGB6*	integrin, beta 6	2:q24.2
*ITGAV*	integrin alpha-V precursor	2:q32.1
*IL6*	interleukin 6 (interferon, beta 2)	7:p15.3
*DEFB1*	defensin, beta 1 preproprotein	8:p23.1
*TLR4*	toll-like receptor 4 precursor	9:q33.1
*MBL2*	soluble mannose-binding lectin precursor	10:q21.1
*SFTPD*	pulmonary surfactant-associated protein D	10:q22.3
*GSTP1*	glutathione transferase	11:q13.2
*MMP1*	matrix metalloproteinase 1 preproprotein	11:q22.2
*MMP12*	matrix metalloproteinase 12 preproprotein	11:q22.2
*ITPR2*	inositol 1,4,5-triphosphate receptor, type 2	12:p11.23
*VDR*	vitamin D (1,25-dihydroxyvitamin D3) receptor	12:q13.11
*DCN*	decorin isoform b precursor	12:q21.33
*PHF11*	PHD finger protein 11	13:q14.3
*SERPINA3*	serpin peptidase inhibitor, clade A, member 3	14:q32.13
*SERPINA1*	serine (or cysteine) proteinase inhibitor, clade	14:q32.13
*IL4R*	interleukin 4 receptor alpha chain isoform b	16:p12.1
*CRHR1*	corticotropin releasing hormone receptor 1	17:q21.31
*AZU1*	azurocidin 1 preproprotein	19:p13.3
*ELA2*	elastase 2, neutrophil preproprotein	19:p13.3
*TGFB1*	transforming growth factor, beta 1	19:q13.2
*IL11*	interleukin 11 precursor	19:q13.42
*ADAM33*	ADAM metallopeptidase domain 33 isoform alpha	20:p13
*MMP9*	matrix metalloproteinase 9 preproprotein	20:q13.12
*SLPI*	secretory leukocyte peptidase inhibitor	20:q13.12

**Table 2:** Anthropometric and clinical characteristics and postural change in blood pressure parameters of the study subjects.

		Discovery cohort (*n* = 480)		Replication cohort (*n* = 465)	
Characteristic	Status	Mean ± SD	No. (%)	Mean ± SD	No. (%)
Age (years)		71 ± 7.3		71.6 ± 7.1	
BMI (m/kg^2^)		28 ± 3.9		27.9 ± 4	
ΔDBP (mmHg)		0.7 ± 6.4		0.5 ± 6.5	
ΔSBP (mmHg)		−2.7 ± 12.9		−1.6 ± 12.5	
PM 48 hr (μg/m^3^)		11.9 ± 6.1		11.8 ± 6	
Smoking	Never		157 (32.71)		143 (30.75)
	Current		25 (5.21)		29 (6.24)
	Former		298 (62.08)		293 (63.01)
Diabetes diagnosis	No		374 (77.92)		351 (75.48)
	Yes		106 (22.08)		114 (24.52)
Alpha blockers	No		393 (81.88)		389 (83.66)
	Yes		87 (18.13)		76 (16.34)
Angiotensin receptor blocker	No		451 (93.96)		430 (92.47)
	Yes		29 (6.04)		35 (7.53)
ACE inhibitors	No		329 (68.54)		334 (71.83)
	Yes		151 (31.46)		131 (28.17)
Calcium channel blockers	No		358 (74.58)		360 (77.42)
	Yes		122 (25.42)		105 (22.58)
Beta blockers	No		287 (59.79)		283 (60.86)
	Yes		193 (40.21)		182 (39.14)

**Table 3:** Characteristics of SNPs with confirmed associations and their distribution among study participants.

Characteristic	*PHF11*	*MMP1*	*ITPR2*	*ITPR2*	*ITPR2*
Rs number	rs9568232	rs1144393	rs16930692	rs7955200	rs10771283
Chromosome	chr13	chr11	chr12	chr12	chr12
Location	48987845	102174619	26477273	26477096	26478845
SNP	C/T	C/T	A/C	C/T	A/G
Functional role	Intron	Promoter	Intron	Intron	Intron
Wild type	814	351	802	480	571
Heterozygote	120	439	119	362	307
Homozygote	3	127	3	82	47
Total	937	917	924	924	925
HWE χ^2^	0.162	1.33	0.16	1.3	0.47
*p*-Value	0.69^a^	0.35	0.70^a^	0.25	0.49

HWE, Hardy–Weinberg equilibrium.

a Yates continuity corrected where expected value < 5.

**Table 4:** Results for ΔSBP SNP × PM_2.5_ interaction models and ARB and ACE inhibitor interactions.^a^

Gene	Variable	Model 1: SNP × 10 μg/m^3^ PM_2.5_ β (95% CI)	Model 2: model 1 + ARB × 10 μg/m^3^ PM_2.5_ β (95% CI)	Model 3: model 1 + ACE inhibitor × 10 μg/m^3^ PM_2._ β (95% CI)
*MMP1*				

Recessive	Rs1144393	0.37 (−1.34 to 2.08)	0.35 (−1.36 to 2.06)	0.38 (−1.33 to 2.09)
	10 μg/m^3^ PM_2.5_	0.2 (−0.80 to 1.20)	0.04 (−0.96 to 1.04)	0.35 (−0.73 to 1.43)
	Rs1144393 × 10 μg/m^3^ PM_2.5_	3.4 (0.79 to 6.01)	3.35 (0.74 to 5.96)	3.46 (0.85 to 6.07)
	ARB		2.76 (−0.57 to 6.09)	
	ARB × 10 μg/m^3^ PM_2.5_		8.64 (2.00 to 15.28)	
	ACE inhibitors			−0.26 (−1.75 to 1.23)
	ACE inhibitors × 10 μg/m^3^ PM_2.5_			−0.83 (−3.20 to 1.54)

*ITPR2*				

Dominant	Rs16930692	−1.07 (−2.75 to 0.61)	−1.06 (−2.74 to 0.62)	−1.07 (−2.75 to 0.61)
	10 μg/m^3^ PM_2.5_	0.28 (−0.72 to 1.28)	0.12 (−0.89 to 1.12)	0.42 (−0.67 to 1.52)
	Rs16930692 × 10 μg/m^3^ PM_2.5_	3.48 (0.84 to 6.12)	3.47 (0.84 to 6.10)	3.45 (0.81 to 6.09)
	ARB		2.89 (−0.45 to 6.23)	
	ARB × 10 μg/m^3^ PM_2.5_		8.68 (2.03 to 15.32)	
	ACE inhibitors			−0.19 (−1.68 to 1.30)
	ACE inhibitors × 10 μg/m^3^ PM_2.5_			−0.74 (−3.12 to 1.63)
Recessive	Rs7955200	−0.73 (−2.77 to 1.30)	−0.75 (−2.78 to 1.29)	−0.74 (−2.77 to 1.30)
	10 μg/m^3^ PM_2.5_	0.33 (−0.63 to 1.29)	0.18 (−0.79 to 1.15)	0.44 (−0.63 to 1.51)
	Rs7955200 × 10 μg/m^3^ PM_2.5_	4.84 (1.47 to 8.20)	4.65 (1.29 to 8.01)	4.79 (1.42 to 8.16)
	ARB		2.87 (−0.47 to 6.20)	
	ARB × 10 μg/m^3^ PM_2.5_		8.35 (1.69 to 15.01)	
	ACE inhibitors			−0.3 (−1.79 to 1.19)
	ACE inhibitors × 10 μg/m^3^ PM_2.5_			−0.58 (−2.95 to 1.80)
Recessive	Rs10771283	−1.29 (−3.82 to 1.23)	−1.29 (−3.82 to 1.23)	−1.3 (−3.83 to 1.23)
	10 μg/m^3^ PM_2.5_	0.41 (−0.55 to 1.36)	0.26 (−0.70 to 1.22)	0.51 (−0.55 to 1.57)
	Rs10771283 × 10 μg/m^3^ PM_2.5_	5.7 (1.71 to 9.69)	5.43 (1.44 to 9.41)	5.63 (1.63 to 9.63)
	ARB		2.88 (−0.46 to 6.21)	
	ARB × 10 μg/m^3^ PM_2.5_		8.24 (1.58 to 14.90)	
	ACE inhibitors			−0.31 (−1.81 to 1.18)
	ACE inhibitors × 10 μg/m^3^ PM_2.5_			−0.54 (−2.91 to 1.84)

a Models adjusted for age, BMI, smoking status, ACE inhibitors, ARBs, diabetes, alpha blockers, beta blockers, and calcium channel blockers. β is equivalent to mmHg.
